# Splenic torsion in polysplenia syndrome: a rare cause of acute abdomen in a paediatric patient

**DOI:** 10.1093/jscr/rjaf771

**Published:** 2025-10-03

**Authors:** Shaima Osman Mohamed Ali Alaraby, Sara Y Mukhtar, Mohamed Y Ibrahim, Eman Mohamed, Shima Abdalraheem Elnadeef Hussein, Mohamed Osman Mohamed Idres

**Affiliations:** Department of Paediatric Surgery, Elmek Nimir University Hospital, Shendi 45512, River Nile State, Sudan; Department of Paediatric Surgery, Elmek Nimir University Hospital, Shendi 45512, River Nile State, Sudan; Paediatric Surgery Center, National Ribat University Hospital, Burri, Khartoum 11111, Sudan; Department of General Surgery, Elmek Nimir University Hospital, Shendi 45512, River Nile State, Sudan; Department of Emergency Medicine, Alhakeem Specialized Hospital, Al-Molazmeen St, Al-Shuhada, Khartoum 14415, Sudan; Department of Anatomy, Sudan International University, Obaid Khatim Street, Arkawait, Khartoum 11111, Sudan

**Keywords:** polysplenia syndrome, accessory spleen, splenic torsion, heterotaxy syndrome

## Abstract

Acute abdominal pain in children presents a significant diagnostic challenge due to its broad differential, including rare congenital anomalies. Torsion of an accessory spleen is a rare but important cause, particularly in the context of polysplenia syndrome. We report the case of a 6-year-old boy with polysplenia syndrome who presented with severe abdominal pain, vomiting, and diarrhea. Imaging revealed multiple splenic masses, and exploratory laparotomy identified a torsed accessory spleen with a twisted vascular pedicle. Resection of the infarcted splenule and correction of associated intestinal malrotation were performed. The patient recovered uneventfully and was discharged on the third postoperative day. Splenic torsion in polysplenia syndrome should be considered in the differential diagnosis of acute abdomen in children with congenital anomalies. Prompt imaging and surgical intervention are essential for favourable outcomes, and increased awareness may aid in early diagnosis and management of this rare condition.

## Introduction

Acute abdominal pain in children presents a significant diagnostic challenge due to its broad differential, ranging from common conditions such as appendicitis to rare congenital anomalies. Among these rare causes, torsion of an accessory spleen or wandering spleen is particularly noteworthy, especially in the context of congenital splenic malformations such as polysplenia syndrome [1–4].

Polysplenia syndrome is a rare and complex congenital anomaly, classified as a form of left isomerism variant within the spectrum of heterotaxy syndrome (situs ambiguous) [4, 5]. It is characterized by the presence of multiple spleens in association with other congenital anomalies including cardiac anomalies, vascular malformations and gastrointestinal anomalies such as intestinal malrotation and biliary atresia. It has variable clinical presentations, often asymptomatic with incidental discovery during imaging or surgery [5]; however, torsion of these splenules can lead to acute abdominal pain, infarction, hemorrhage, or peritonitis [1, 3, 6–8]. The clinical presentation is often non-specific, mimicking more common causes of acute abdomen and frequently resulting in delayed diagnosis [1]. Imaging modalities such as abdominal ultrasound and contrast-enhanced computed tomography (CT) scans can aid in diagnosis, but definitive identification is often made intraoperatively [1]. Recent case reports highlight the importance of considering splenic torsion in children with acute abdominal symptoms, particularly those with congenital anomalies or malrotation [4, 9–13].

Here, we present a rare case of splenic torsion in a child with polysplenia syndrome, highlighting the diagnostic challenges and emphasizing the need for prompt surgical management in such presentations.

## Case report

**Figure 1:**
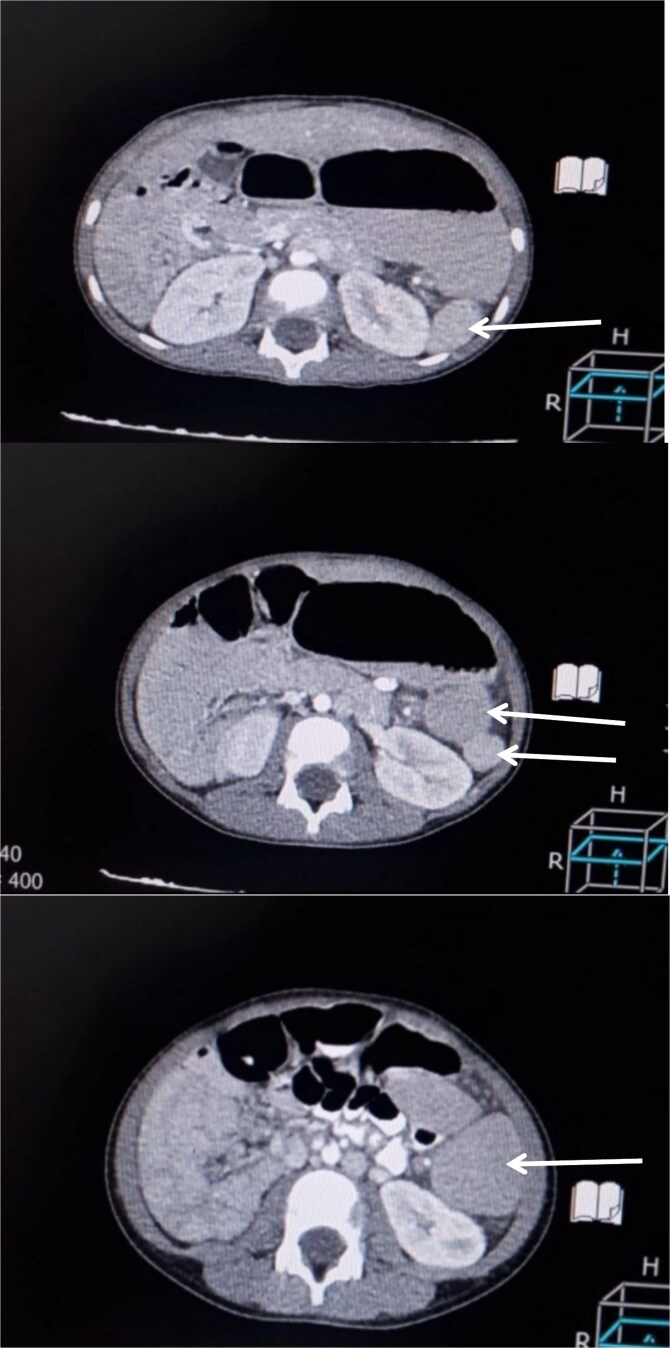
Contrast-enhanced CT scan of the abdomen demonstrating multiple spleen-like densities (arrows) can be seen in the left upper and lower quadrants of the abdomen.

A 6-year-old boy presented with a 2-day history of abdominal pain, vomiting, and diarrhea. The pain was of sudden onset, severe, intermittent, mainly in the umbilical region, not radiating anywhere, with no aggravating or relieving factors. It was associated with non- bilious vomiting of small amounts of food contents, in addition to small amounts of diarrhea not containing mucus or blood. Upon admission, the patient looked unwell but not febrile. His pulse rate was 100 beats/min, and the respiratory rate was 24 breaths/min. Abdominal examination revealed a firm, non-tender mass, ~8 × 4 cm in diameter with a smooth surface, palpable just left of the umbilicus. Laboratory investigations were unremarkable. An abdominal ultrasound was performed initially that showed a well-defined, 10 × 4 cm homogenous mass, seen at the midline and extending to the left of the midline suggestive of an enlarged spleen. As the ultrasound was inconclusive, a contrast-enhanced CT abdomen was obtained, which demonstrated multiple spleen-like densities in the left upper and lower abdominal quadrants with minimal free fluid in the left iliac fossa (Fig. 1).

**Figure 2:**
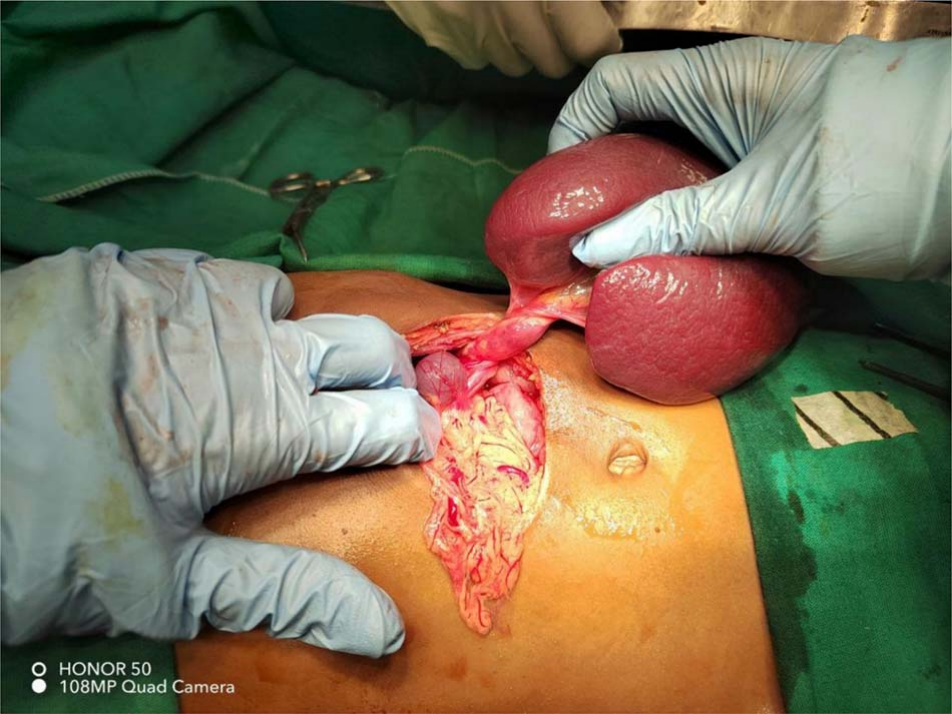
Intraoperative photograph showing an accessory spleen with a twisted long vascular pedicle, consistent with torsion.

**Figure 3:**
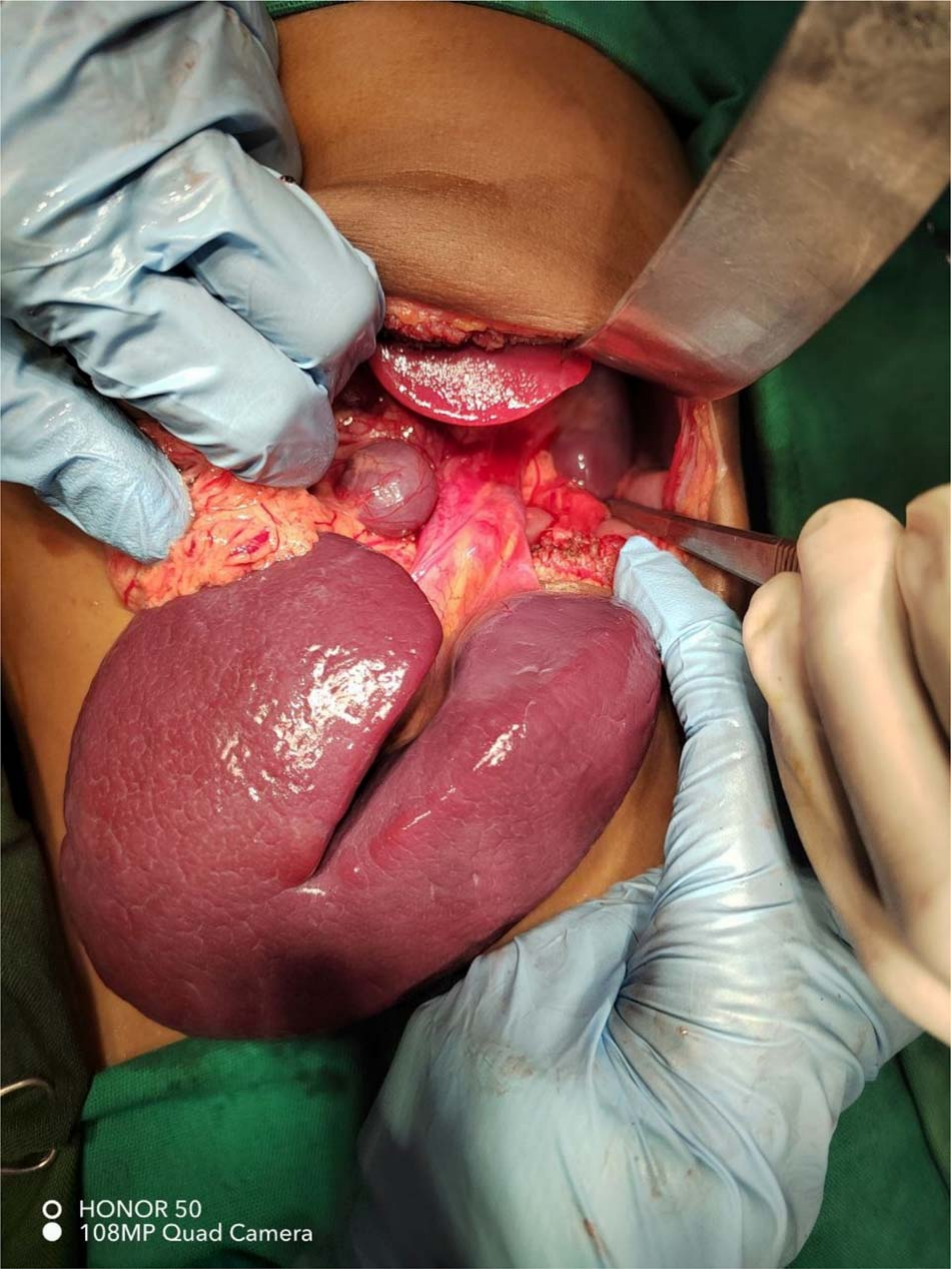
Intraoperative photograph showing two accessory spleens: A large fissured spleen with a long vascular pedicle and a smaller rounded splenule attached to the omentum. The normal spleen is seen in the left upper quadrant of the abdomen.

In view of the unavailability of laparoscopy, an exploratory laparotomy was performed with a preoperative suspicion of accessory splenic torsion. Through a transverse supra-umbilical incision, a large violet mass with a twisted long vascular pedicle was identified, consistent with accessory splenic torsion (Fig. 2). Detorsion and resection of the mass were performed. Two other small splenules were noted; one within the greater omentum and another within the mesentery (Fig. 3), and were left in situ. The native spleen was present in its normal anatomical position. Further exploration revealed malrotation; the DJ flexure was located on the right side and the cecum was on the left side of the abdomen. Therefore, a Ladd’s procedure was performed.

The post-operative course was uneventful; oral intake was resumed on post-operative Day 2, and the patient was discharged home on Day 3 in a good condition. Histological examination confirmed splenic tissue with an intact capsule and no abnormalities of histological structures.

## Discussion

Splenic torsion in the setting of polysplenia syndrome represents an unusual but important cause of acute abdominal pain in paediatric patients. The diagnostic workup is typically complex secondary to the non-specific nature of the presentation and potential for overlapping with common abdominal diseases. These patients present with symptoms in the form of abdominal pain and vomiting, and rarely with diarrhea, but in a manner that may lead to misdiagnosis as gastroenteritis or appendicitis. During the physical examination in the case at hand, the finding of a firm but non-tender abdominal mass increased suspicion for an atypical etiology; however, the initial laboratory tests were ultimately non-diagnostic in accordance with the current literature stating that hematologic studies often do not provide characteristic alterations [1–4, 13].

Imaging modalities play a central part in the diagnostic workup. While ultrasound can suggest the presence of a mass or splenomegaly, contrast-enhanced CT is best suited to describe accessory spleens and signs of torsion in the form of a twisted vascular pedicle or an infarcted spleen [1–4, 13]. A definitive diagnosis, however, is often made intraoperative, as in this case where exploratory laparotomy revealed a twisted and infarcted splenule with an associated long vascular pedicle. The surgery involved detorsion and resection of the involved splenule with preservation of the intact accessory spleens and the native spleen in accordance with modern guidelines to avoid the risk of overwhelming post-splenectomy infection, particularly in children [1–4, 8, 13].

An important aspect highlighted in this case relates to the frequent association of polysplenia syndrome with other congenital anomalies, particularly gastrointestinal system anomalies. The intraoperative finding of intestinal malrotation in this patient, which was treated by a Ladd’s procedure, points toward the importance of an extensive evaluation in cases of confirmed or suspected polysplenia because a variety of anomalies may be present and affect presentation as well as the treatment course [4, 9–13].

The current body of literature attests to the importance of sustaining an increased state of alertness for unusual causes of acute abdominal pain in children and adolescents, in particular in cases where congenital anomalies were determined or when clinical presentation varies from predicted standards. The timely use of modern imaging modalities is important because such modalities can offer clarity in terms of anatomic aberrations and aid in planning surgical intervention. There is a need for prompt surgical intervention in cases of torsion of the spleen since delay may lead to serious complications like infarction, peritonitis, or sepsis. The uncomplicated postoperative recovery and histopathologic confirmation of a normal spleen in this presentation serve as an example of the favourable outcome associated with early identification and intervention [1–4, 6–13].

Splenic torsion has to always be included in the differential diagnosis of acute conditions in children with congenital anomalies like polysplenia syndrome, state Ahmed et al. The unclear nature of clinical presentation in many instances often leads to delay in diagnoses; however, imaging modalities—particularly contrast-enhanced CT—are the key to establishing a preoperative diagnosis. The clarification of the diagnosis and treatment in most instances fall to surgical exploration, and timely intervention serves to prevent complications like infarction, peritonitis, or sepsis [13].

In conclusion, splenic torsion associated with polysplenia syndromes, though rare, should be included in the differential diagnoses of acute abdominal processes in children, particularly those with congenital malformations. Prompt diagnosis, the use of appropriate imaging studies, and early surgical intervention are the keys to obtaining good clinical outcomes. Increased awareness among clinicians and further research will be instrumental in formulating adequate diagnostic and therapeutic protocols for this rare but potentially severe condition [1–4, 6–13].
